# Barriers affecting the quality and consistency of barium studies in radiologists and registrars

**DOI:** 10.1007/s00261-024-04791-x

**Published:** 2025-01-13

**Authors:** Muhammad Faraz Mangi, Mohammad Danish Mangi, WanYin Lim

**Affiliations:** 1 University of Adelaide, Adelaide, Australia; 2 SA Health, Adelaide, Australia; 3 Jones Radiology, Adelaide, Australia

**Keywords:** Barium swallow, Gastrointestinal tract, Deglutition disorders, Abdomen, Radiology

## Abstract

The barium swallow study is a fluoroscopic study which provides valuable insights into the motility, function and morphology of the pharynx, oesophagus, gastroesophageal junction, proximal stomach and duodenum. It has been observed that the skill of radiology doctors with barium swallow studies in adults has diminished. This reduced proficiency with barium swallow study is closely linked to and perpetuated by the heterogeneity of technique amongst radiologists. Factors pertaining to the individual radiologist, patient factors, healthcare factors, and the widespread use of alternative investigations have led to this increased variance in performing the barium swallow study. Despite this reduction in its usage, the study remains a valuable tool in the care of patients. We advocate for standardised guidelines to increase consistency and improve radiologist familiarity with this procedure.

## Introduction

The barium swallow study is a fluoroscopic study which provides valuable insights into the motility, function and morphology of the pharynx, oesophagus, gastroesophageal junction, proximal stomach and duodenum [1]. Due to various factors, it is observed that the skill of radiology doctors with adult barium swallow studies has diminished. This reduced proficiency with barium swallow study is closely linked to and perpetuated by the heterogeneity of technique amongst radiologists [2]. Despite this reduction in its usage, the study remains a valuable tool in the care of patients [3]. We discuss the few factors that may have led to the current situation. We advocate for standardised Australian guidelines in performing adult barium swallow studies and for radiology teaching to be consistent, regardless of limitations such as supply shortages or heterogeneity in ordering of procedures.

## Radiologist factors

The range of barium swallow techniques is wide. Radiologists aim to tailor the study towards the clinical indication [4]. This includes the use of different views, projections, patient positions, and density of barium preparations [5]. Moreover, differences in fluoroscopy machinery influence radiologist technique. Differences in the position of the X-ray tube may affect the views that can be taken. As the technology progresses, machines may have faster frame or acquisition rates, as well as reductions in radiation dose per study, changes that not all radiologists may know about [6]. Terminology used to report this investigation is also not standardised and poorly defined, thus creating variability in reporting styles [7]. In addition, there is a lack of normative data for barium swallow studies as control data is limited [7]. As such, interpretation for barium swallow can become largely subjective, with overlap of normal physiological findings at different ages as well as diseases [8].

At the moment, radiology training for barium swallow technique is limited. There is brief formal teaching and no standardisation in technique for barium swallow in Australia, with a lack of guidelines [9]. As such, alternative resources are used as guides, such as the Radiopaedia reference article, the British Society of Gastrointestinal and Abdominal Radiology barium swallow webpage, and the Giesel School of Medicine Fluoroscopy articles [10–12]. Whilst these guides are valid for performing the barium swallow study and likely contain content similar to a potential future Australian guideline, they offer subtle differences in positioning and frames taken. Moreover, this lack of formal Australian guidelines creates confusion amongst radiology doctors in choosing which guide to adhere to, with many practitioners instead relying on past teaching and experience.

The culture of radiology teaching for procedures, like other medical fields such as surgery and emergency medicine, is characterised by the adage “see one, do one, teach one” or in other terms, consultant-lead observation and training for technique, and then confirmation of competency with supervision [13]. This can lead to registrars only being exposed to the methods used by their consultants. Over time, issues with barium swallow technique, such as unfavourable shortcuts, can compound as new registrars are trained by new consultants. In tandem, there is subjectivity in interpretation, dependent on the individual radiologist or institution expertise [2]. This has been cited as an issue in other countries as well, with American residency programs increasingly having a reduced focus on performing the barium swallow study, leading to new radiologists having reduced familiarity with the investigation [5]. Additionally, global factors, such as the COVID-19 pandemic have had a major impact on radiology training, with registrars exposed to fewer barium swallow studies as a result of a reduction in face-to-face teaching for investigation techniques [14].

### Patient factors

Patient factors could contribute to the variety in barium swallow technique. Some patients are frail, have limited mobility, or may be at increased risk of aspiration, affecting the length of the study and the number of projections that can be completed for them [15]. There are instances where the study can only be performed lying down, or in a wheelchair [16]. Additionally, side effects such as nausea, vomiting and stomach cramps associated with the barium drink can cause some patients to become uncomfortable, affecting technique in which these images are taken and potentially, reducing the duration in which these images can be taken [1]. Moreover, some patients may avoid the study due to these short-term side effects as well as the risk of constipation after the investigation is performed, reducing the number and hence, familiarity of radiologists with barium swallow studies [17]. In these cases, alternative investigations, such as a computed tomography (CT) scan of the abdomen are frequently used.

## Healthcare factors

Healthcare factors, such as the recent shortage of barium in Australia, as well as shortages globally in the past remain a significant barrier towards a more widespread usage of and familiarity with the barium swallow study [18–20]. With the cost of the study rising and availability reducing, other investigations for oesophageal pathology, such as endoscopy and oesophageal manometry, have been used as alternatives to help conserve the stores of barium [21]. Since lesions noted on a barium swallow study typically need to be assessed further via endoscopy as well as the fact that intervention is possible with endoscopy, it has become more common for endoscopy to become an initial investigation for most symptoms, except for motility disorders. To still perform the barium swallow study, low density bariums, Ultravist (iodinated contrast), and Gastrografin, have been used as substitutes. Whilst these alternatives allow motility assessment, their limited ability to coat the oesophagus leads to an inadequate assessment of the mucosal lining [22]. Additionally, there is also a shortage in the effervescent used in barium swallow studies. Radiologists have had to use alternatives such as carbonated drinks to dilate the stomach, albeit to variable results. Whilst these shortages have only persisted for around 2 years, this constitutes a significant amount of a trainee time, resulting in graduating registrars having had minimal exposure or experience in performing barium swallow [18]. Similar to how in the United States of America, the barium swallow study has seen a decrease in usage due to a lack of experienced radiologists, this is likely to have a sustained effect downstream, affecting registrar training as future teaching consultants may have limited expertise in this investigation [21].

Despite its reduced use, the barium swallow study remains a valuable tool and an important skill for radiologists and radiology registrars to master and teach. Whilst having similar uses, the barium swallow study does hold some advantages over endoscopy in certain scenarios. The barium swallow study is non-invasive and associated with milder side effects, in comparison to endoscopy which can cause irritation, bleeding or even perforation of the gastrointestinal tract [23]. Moreover, the barium swallow study remains highly useful for oesophageal motility disorders. For example, achalasia can appear normal on an endoscopy, whereas the classical “bird beak” sign can be clearly identified on barium swallow [24]. In addition to being used as a primary investigation, once the barium and effervescent shortage is addressed, there is also potential for the barium swallow to be used as a relatively safe and quick screening tool for patients with upper gastrointestinal symptoms, such as reflux and dysphagia, in order to help with triaging those who may require urgent gastroenterology review and further investigations such as endoscopy or colonoscopy [25].

## Conclusion

Factors pertaining to the individual radiologist, patient factors, healthcare factors, and the widespread use of alternative investigations have led to increased heterogeneity in technique for the barium swallow study. Despite this, the barium swallow study remains a valuable diagnostic and screening tool for adults and hence, we advocate for Australian standardised technique guidelines. These would include a standardised method to performing the barium swallow study, as well as providing approaches to performing this study in patient populations where standard views cannot be taken, due to issues with positioning or mobility. These would increase consistency among radiology doctors, improve familiarity and ensure that essential projections and assessments are not omitted.

## Data Availability

No datasets were generated or analysed during the current study.
